# Supplementary material for: RBP-Driven RNA sorting and local translation establish the molecular identities of individual sensory axons
Source: Nat Commun. 2026 Aug 31;17:10345. doi: 10.1038/s41467-026-77192-x (PMC13623950; doi:10.1038/s41467-026-77192-x)
Supplement: Supplementary file 1 [file 41467_2026_77192_MOESM1_ESM.pdf]

Supplementary Information File

Figure S1: TRAP-Seq Experimental Validation.

Figure S2: Functional comparison of DRG soma and axonal translatomes.

Figure S3: Puromycin incorporation into P7 and 2-mo-old axons.

Figure S4: Local translation of ion channels in adult mice.

Figure S5: Validation of mRNA expression of additional transcripts from translatomic datasets.

Figure S6: RNA Binding Protein motif analyses and localization.

Figure S7: SFPQ and SRSF10 RIP-Sequencing.

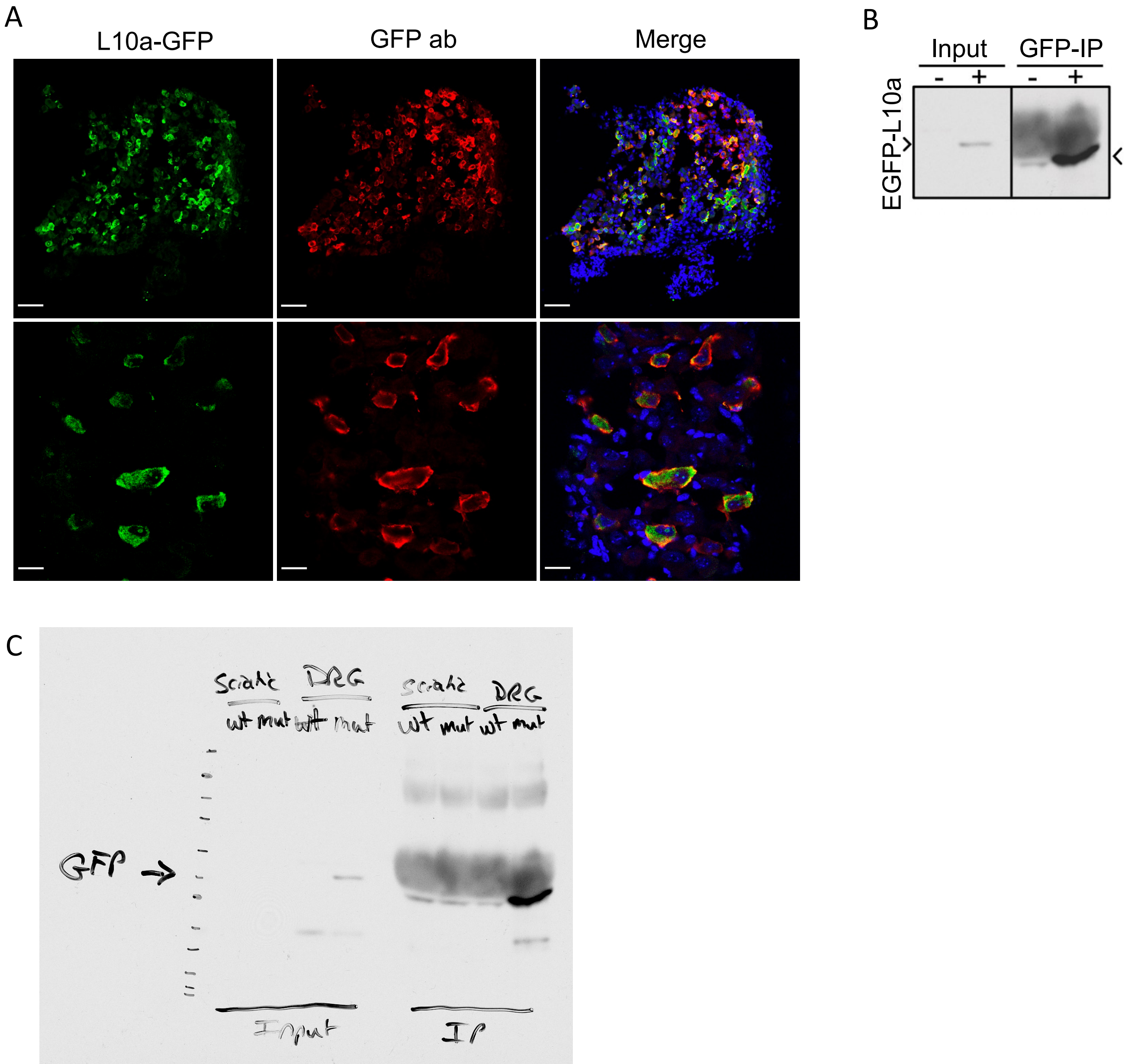

Figure S1: TRAP-Seq Experimental Validation. A) Endogenous L10a-EGFP expression is visible in the soma of DRG neurons from Nav1.8Cre;L10a-EGFPfl/fl mice and can be efficiently targeted and labeled by anti-GFP antibodies (scale bars: 40 µm (top row), 20 µm (bottom row). B, C). Cropped (B) and uncropped Western blot images (C) showing L10a-EGP protein can be immunoprecipitated from tissue lysates with anti-GFP antibodies.

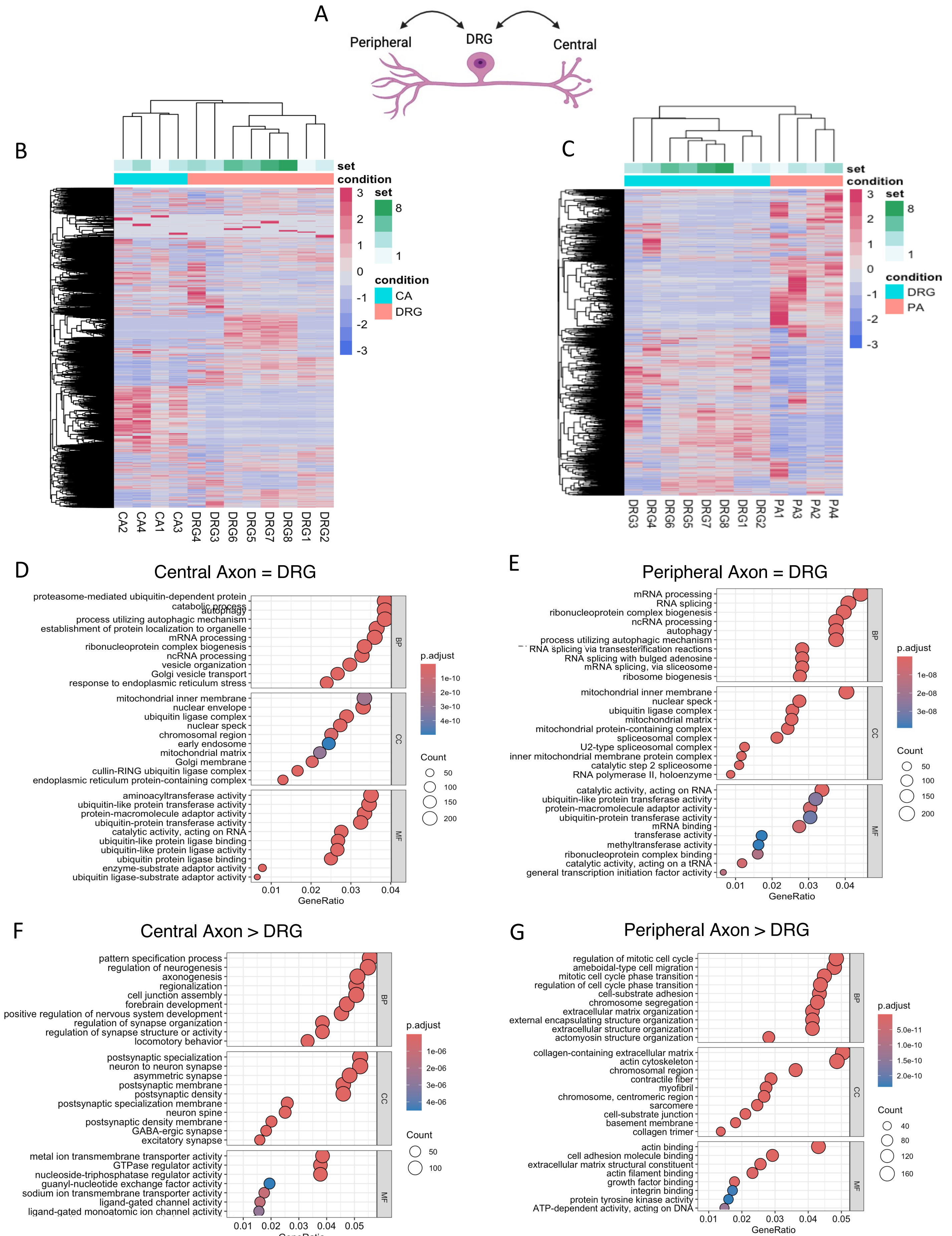

A
Peripheral
DRG
Central
B
C
set
condition
CA
DRG
PA
D
Central Axon = DRG
E
Peripheral Axon = DRG
F
Central Axon > DRG
G
Peripheral Axon > DRG
p.adjust
Count
GeneRatio
BP
CC
MF
proteasome-mediated ubiquitin-dependent protein catabolic process
autophagy
process utilizing autophagic mechanism
establishment of protein localization to organelle
mRNA processing
ribonucleoprotein complex biogenesis
ncRNA processing
vesicle organization
Golgi vesicle transport
response to endoplasmic reticulum stress
mitochondrial inner membrane
nuclear envelope
ubiquitin ligase complex
nuclear speck
chromosomal region
early endosome
mitochondrial matrix
Golgi membrane
cullin-RING ubiquitin ligase complex
endoplasmic reticulum protein-containing complex
aminoacyltransferase activity
ubiquitin-like protein transferase activity
protein-macromolecule adaptor activity
ubiquitin-protein transferase activity
catalytic activity, acting on RNA
ubiquitin-like protein ligase binding
ubiquitin-like protein ligase activity
ubiquitin protein ligase binding
enzyme-substrate adaptor activity
ubiquitin ligase-substrate adaptor activity
mRNA processing
RNA splicing
ribonucleoprotein complex biogenesis
ncRNA processing
autophagy
process utilizing autophagic mechanism
RNA splicing via transesterification reactions
RNA splicing with bulged adenosine
mRNA splicing, via sliceosome
ribosome biogenesis
mitochondrial inner membrane
nuclear speck
ubiquitin ligase complex
mitochondrial matrix
mitochondrial protein-containing complex
spliceosomal complex
U2-type spliceosomal complex
inner mitochondrial membrane protein complex
catalytic step 2 spliceosome
RNA polymerase II, holoenzyme
catalytic activity, acting on RNA
ubiquitin-like protein transferase activity
protein-macromolecule adaptor activity
ubiquitin-protein transferase activity
mRNA binding
transferase activity
methyltransferase activity
ribonucleoprotein complex binding
catalytic activity, acting on a tRNA
general transcription initiation factor activity
pattern specification process
regulation of neurogenesis
axonogenesis
regionalization
cell junction assembly
forebrain development
positive regulation of nervous system development
regulation of synapse organization
regulation of synapse structure or activity
locomotory behavior
postsynaptic specialization
neuron to neuron synapse
asymmetric synapse
postsynaptic membrane
postsynaptic density
postsynaptic specialization membrane
neuron spine
postsynaptic density membrane
GABA-ergic synapse
excitatory synapse
metal ion transmembrane transporter activity
GTPase regulator activity
nucleoside-triphosphatase regulator activity
guanyl-nucleotide exchange factor activity
sodium ion transmembrane transporter activity
ligand-gated channel activity
ligand-gated monoatomic ion channel activity
regulation of mitotic cell cycle
ameboidal-type cell migration
mitotic cell cycle phase transition
regulation of cell cycle phase transition
cell-substrate adhesion
chromosome segregation
extracellular matrix organization
external encapsulating structure organization
extracellular structure organization
actomyosin structure organization
collagen-containing extracellular matrix
actin cytoskeleton
chromosomal region
contractile fiber
myofibril
chromosome, centromeric region
sarcomere
cell-substrate junction
basement membrane
collagen trimer
actin binding
cell adhesion molecule binding
extracellular matrix structural constituent
actin filament binding
growth factor binding
integrin binding
protein tyrosine kinase activity
ATP-dependent activity, acting on DNA

Figure S2

Figure S2. Functional comparison of DRG soma and axonal translatomes. A) Schematic depicting the translatomic gene sets compared in Figure S2 (i.e. DRG soma vs. axons). Created in BioRender. Silagi, E. (2026) https://BioRender.com/ra0oidx. B,C) Heatmaps comparing gene expression profiles within DRG soma and central axon samples (B) or peripheral axon samples (C) (n=4 samples/axon group; n=8 samples/DRG soma group). The rows of genes cluster within groups that show similar expression profiles between soma and axons, and well as groups that have higher expression in either the soma or axon samples. D,E) Gene ontology (GO) enrichment analysis visualized as a dot plot, where each dot represents a significantly enriched biological process (BP), cellular component (CC), or molecular function (MF). These dot plots show the GO terms enriched among the genes that are shared by both the DRG soma translatome and the central axon translatome (D) or peripheral axon translatome (E) (p.adj $\geq$ 0.05; abs[F.C] $\leq$ 1.5). F, G) These dot plots show GO terms that are enriched among the genes with higher expression in the central axon translatome (F) or peripheral axon translatome (G) as compared to the DRG soma translatome (p.adj $\leq$ 0.05; F.C $\geq$ -1.5).

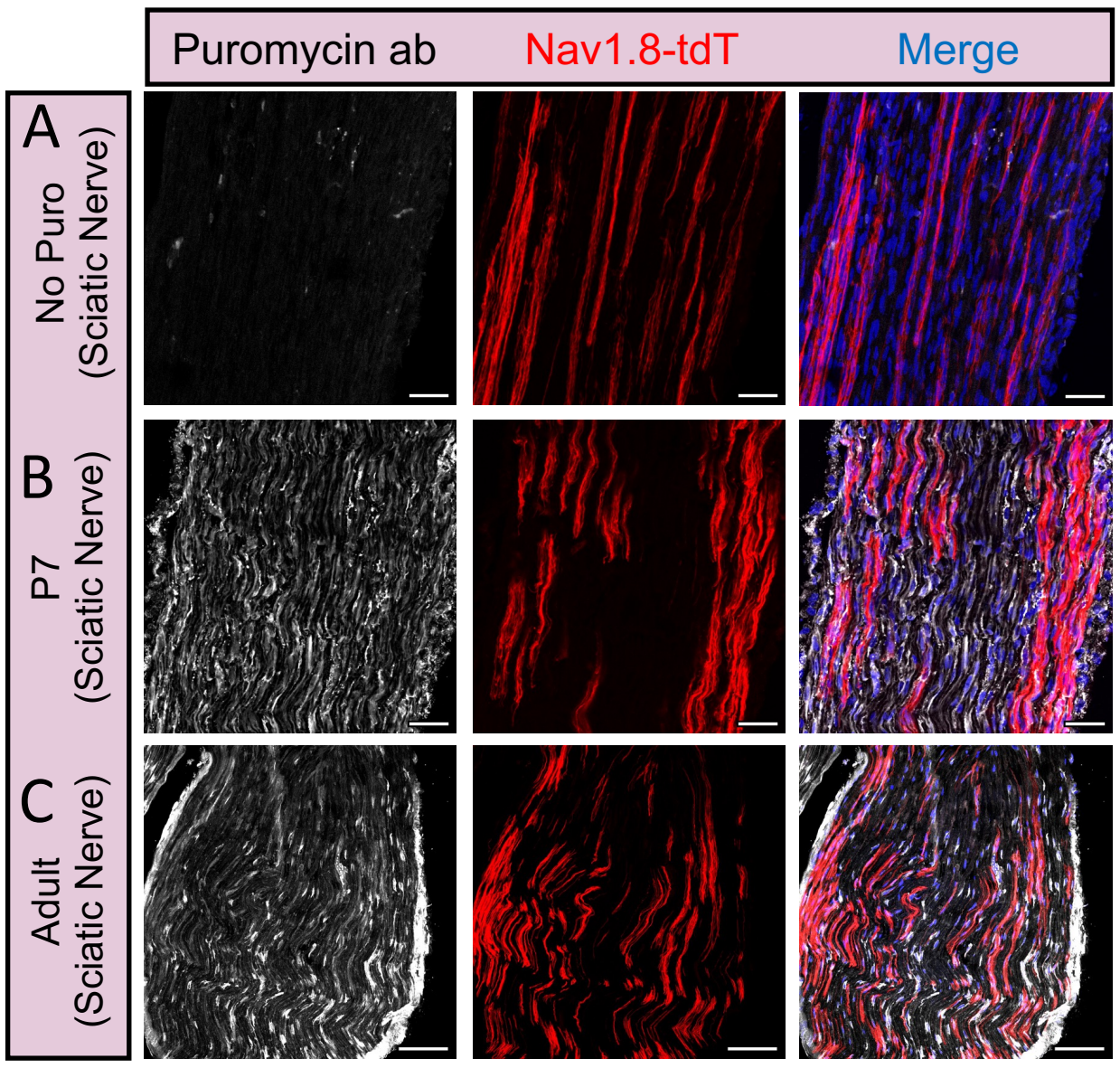

Figure S3. Puromycin incorporation into P7 and 2-mo-old axons. Representative fluorescent immunohistochemistry (IHC) images of anti-puromycin antibody staining (white), TdTomato-labeled Nav1.8+ (Nav1.8-tdT) axons (red), and merged images with DAPI-labeled nuclei (blue) from a P7 negative control sciatic nerve (A), P7 puromycin-treated sciatic nerve (B), and 2-mo-old puromycin-treated sciatic nerve (C). Scale bar = 40 µm (P7); 100 µm (Adult).

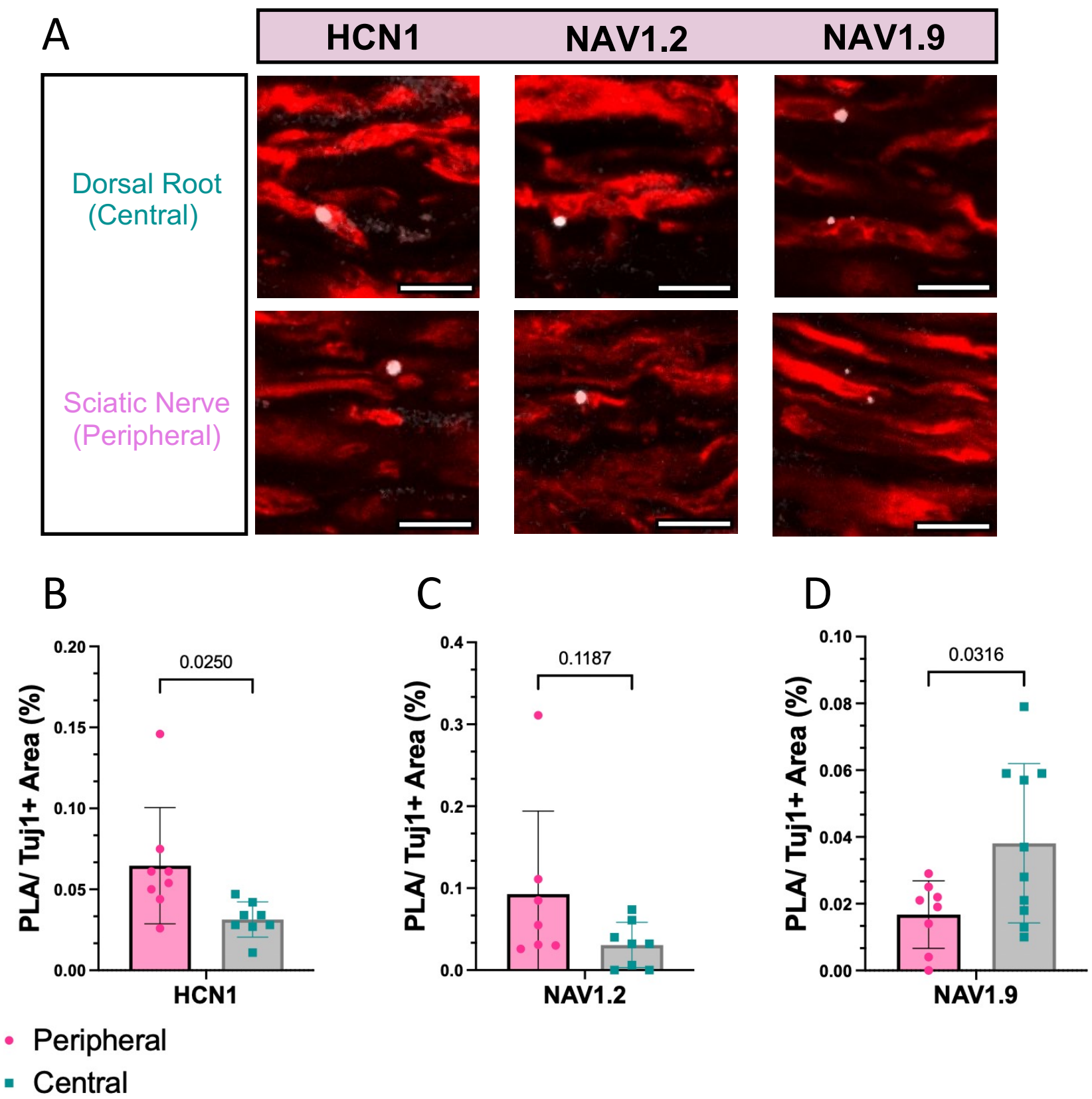

Figure S4. Local translation of ion channels in adult mice. A) Representative high magnification (~120x) images demonstrating the location and abundance of HCN1, NAV1.2, and NAV1.9 Puro-PLA puncta (white) within TUJ1+ axons (red) in the dorsal roots and sciatic nerves of 2-mo-old mice. Scale bar = 10 µm. B) Quantification of the percent area occupied by HCN1 Puro-PLA puncta within TUJ1+ axons in individual dorsal roots (n=8) and sciatic nerves (n=8) of 2-mo-old mice. C) Quantification of the percent area occupied by NAV1.2 Puro-PLA puncta within TUJ1+ axons in individual dorsal roots (n=8) and sciatic nerves (n=7) of 2-mo-old mice. D) Quantification of the percent area occupied by NAV1.9 Puro-PLA puncta within TUJ1+ axons in individual dorsal roots (n=10) and sciatic nerves (n=8) of 2-mo-old mice. Representative images with enhanced brightness shown; raw images used in analysis. Statistical significance determined by two-way ANOVA with Tukey's multiple comparisons test and multiple unpaired t-tests (p-value ≤ 0.05). Data are represented as mean ± SD. All statistical analyses were derived from data using 4 mice. Source data are provided as a Source Data file.

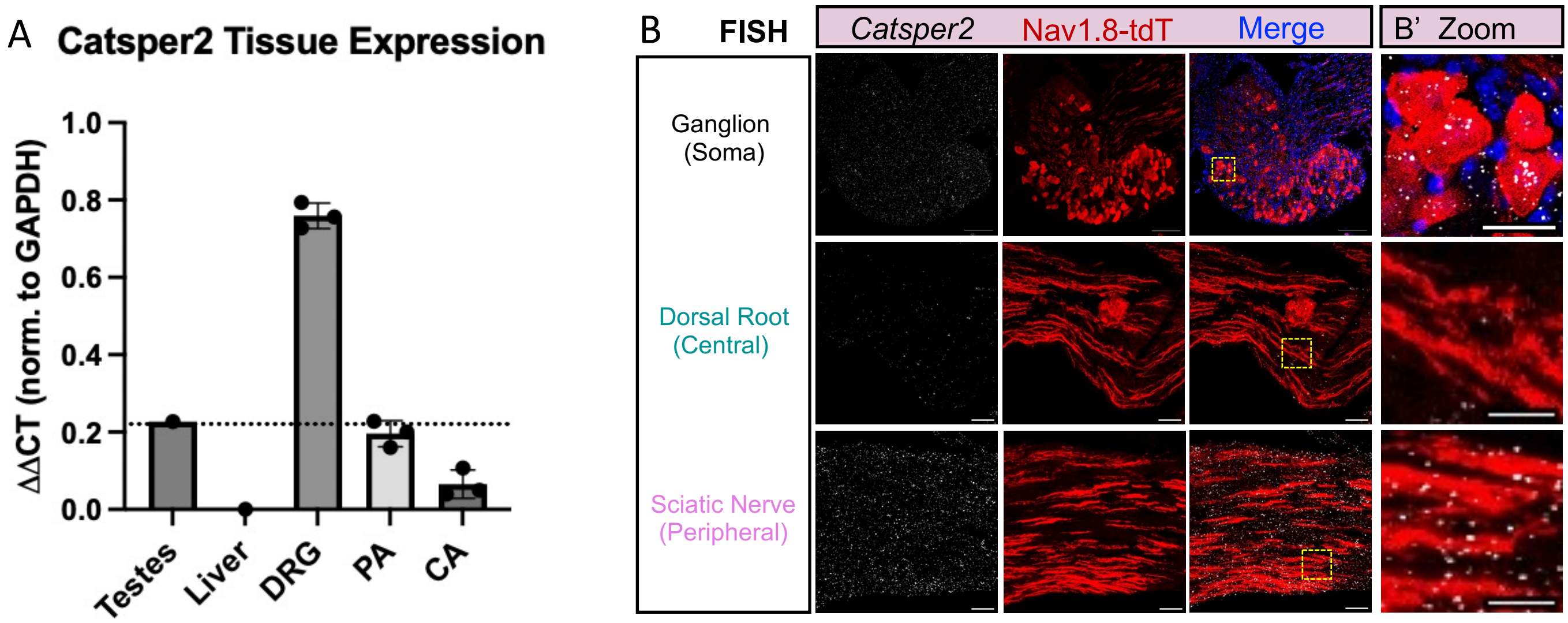

Figure S5. Validation of mRNA expression of additional transcripts from translatomic datasets. A) qRT-PCR data demonstrating relative *Catsper2* mRNA expression in mouse testes (positive control), liver (negative control), DRG (soma), sciatic nerves (peripheral axons), and dorsal roots (central axons). All samples are normalized to GAPDH expression. Data are represented as mean ± SD. B) Representative 20x2 fluorescent images of *Catsper2* mRNA puncta (visualized by RNAScope *in situ* hybridization) and TdTomato-labeled Nav1.8+ (Nav1.8-tdT) neurons in DRGs, dorsal roots, and sciatic nerves, representing DRG soma, central axons, and peripheral axons, respectively. Scale bar = 100 μm (soma) and 40 μm (axons). B') Higher magnification images demonstrating the location and abundance of *Catspser2* mRNA puncta within Nav1.8-tdT+ soma in the ganglia, and axons in the dorsal root and sciatic nerve. Scale bar = 25 μm (soma) and 15 μm (axons). Source data are provided as a Source Data file.

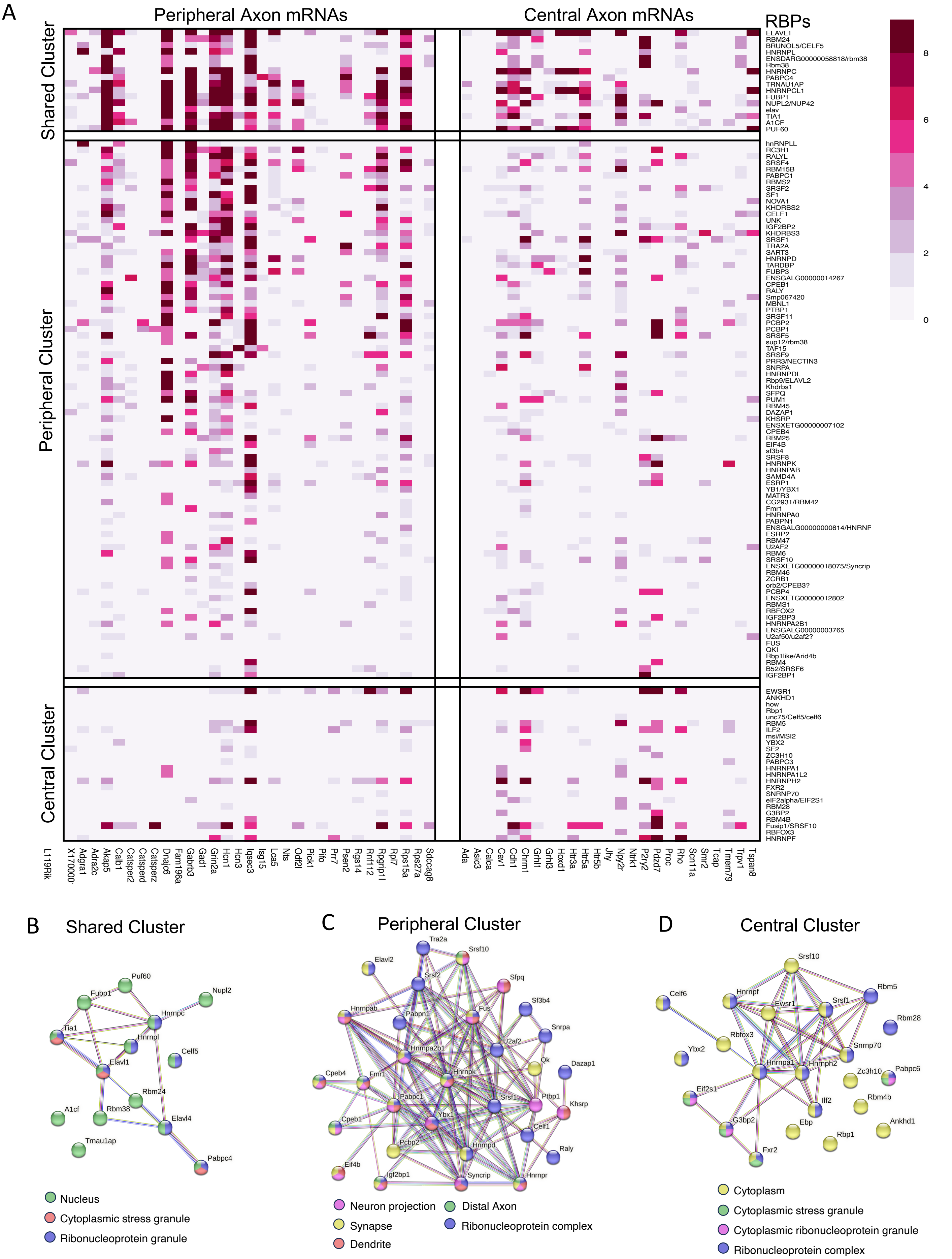

A
Peripheral Axon mRNAs
Central Axon mRNAs
RBPs
Shared Cluster
Peripheral Cluster
Central Cluster
ELAVL1
RBM24
BRUNOL5/CELF5
HNRNPL
ENSDARG00000058818/rbm38
Rbm38
HNRNPC
PABPC4
TRNAU1AP
HNRNPCL1
FUBP1
NUPL2/NUP42
elav
TIA1
A1CF
PUF60
hnRNPLL
RC3H1
RALYL
SRSF4
RBM15B
PABPC1
RBMS2
SRSF2
SF1
NOVA1
KHDRBS2
CELF1
UNK
IGF2BP2
KHDRBS3
SRSF1
TRA2A
SART3
HNRNPD
TARDBP
FUBP3
ENSGALG00000014267
CPEB1
RALY
Smp067420
MBNL1
PTBP1
SRSF11
PCBP2
PCBP1
SRSF5
sup12/rbm38
TAF15
SRSF9
PRR3/NECTIN3
SNRPA
HNRNPDL
Rbp9/ELAVL2
Khdrbs1
SFPQ
PUM1
RBM45
DAZAP1
KHSRP
ENSXETG00000007102
CPEB4
RBM25
EIF4B
sf3b4
SRSF8
HNRNPK
HNRNPAB
SAMD4A
ESRP1
YB1/YBX1
MATR3
CG2931/RBM42
Fmr1
HNRNPA0
PABPN1
ENSGALG00000000814/HNRNF
ESRP2
RBM47
U2AF2
RBM6
SRSF10
ENSXETG00000018075/Syncrip
RBM46
ZCRB1
orb2/CPEB3?
PCBP4
ENSXETG00000012802
RBMS1
RBFOX2
IGF2BP3
HNRNPA2B1
ENSGALG00000003765
U2af50/u2af2?
FUS
QKI
Rbp1like/Arid4b
RBM4
B52/SRSF6
IGF2BP1
EWSR1
ANKHD1
how
Rbp1
unc75/Celf5/celf6
RBM5
ILF2
msi/MSI2
YBX2
SF2
ZC3H10
PABPC3
HNRNPA1
HNRNPA1L2
HNRNPH2
FXR2
SNRNP70
eIF2alpha/EIF2S1
RBM28
G3BP2
RBM4B
Fusip1/SRSF10
RBFOX3
HNRNPF
8
6
4
2
0
L119Rik
X170000
Adgra1
Adra2c
Akap5
Calb1
Catsper2
Catsperd
Catsperz
Dnajc6
Fam196a
Gabrb3
Gad1
Grin2a
Hcn1
Hcn3
Iqsec3
Isg15
Lca5
Nts
Odf2l
Pick1
Pifo
Prr7
Psen2
Rgs14
Rnf112
Rpgrip1l
Rpl7
Rps15a
Rps27a
Sdccag8
Ada
Asic3
Calca
Cav1
Cdh1
Chrm1
Grhl1
Grhl3
Hoxd1
Htr3a
Htr5a
Htr5b
Jhy
Npy2r
Ntrk1
P2ry2
Pdzd7
Proc
Rho
Scn11a
Smr2
Tcap
Tmem79
Trpv1
Tspan8
B
Shared Cluster
Puf60
Fubp1
Nupl2
Hnrnpc
Tia1
Hnrnpl
Celf5
Elavl1
Rbm24
A1cf
Rbm38
Elavl4
Trnau1ap
Pabpc4
Nucleus
Cytoplasmic stress granule
Ribonucleoprotein granule
C
Peripheral Cluster
Tra2a
Srsf10
Elavl2
Srsf2
Sfpq
Sf3b4
Hnrnpab
Pabpn1
Fus
Snrpa
U2af2
Hnrnpa2b1
Qk
Dazap1
Cpeb4
Fmr1
Hnrnpk
Pabpc1
Srsf1
Ptbp1
Khsrp
Cpeb1
Ybx1
Celf1
Pcbp2
Hnrnpd
Raly
Eif4b
Igf2bp1
Syncrip
Hnrnpr
Neuron projection
Distal Axon
Synapse
Ribonucleoprotein complex
Dendrite
D
Central Cluster
Srsf10
Celf6
Hnrnpf
Rbm5
Ewsr1
Srsf1
Rbm28
Rbfox3
Ybx2
Snrnp70
Hnrnpa1
Hnrnph2
Zc3h10
Pabpc6
Eif2s1
Ilf2
Rbm4b
G3bp2
Ebp
Ankhd1
Rbp1
Fxr2
Cytoplasm
Cytoplasmic stress granule
Cytoplasmic ribonucleoprotein granule
Ribonucleoprotein complex

Figure S6

Figure S6. RNA Binding Protein motif analyses and localization. A) Heatmap displaying the number of RBP motifs within the 3'UTR of mRNAs with axon-preferential translation. RBPs identified via oRNAment, RNAmap, and RPS.renlab. B-D) STRING motif analysis of the cluster of RBPs found to have 3’UTR binding sites in: both central and peripherally targeted transcripts, i.e. “shared cluster” (B), centrally targeted transcripts, i.e. “central cluster” (C), and peripherally targeted transcripts, i.e. “peripheral cluster” (D). Data are represented as protein interaction networks with a high-confidence score of 0.700. Nodes represent individual proteins and are color-coordinated by enriched GO cellular components (FDR $\leq$ 0.05). Edges represent protein-protein associations based on known interactions, predicted interactions, and protein homology.

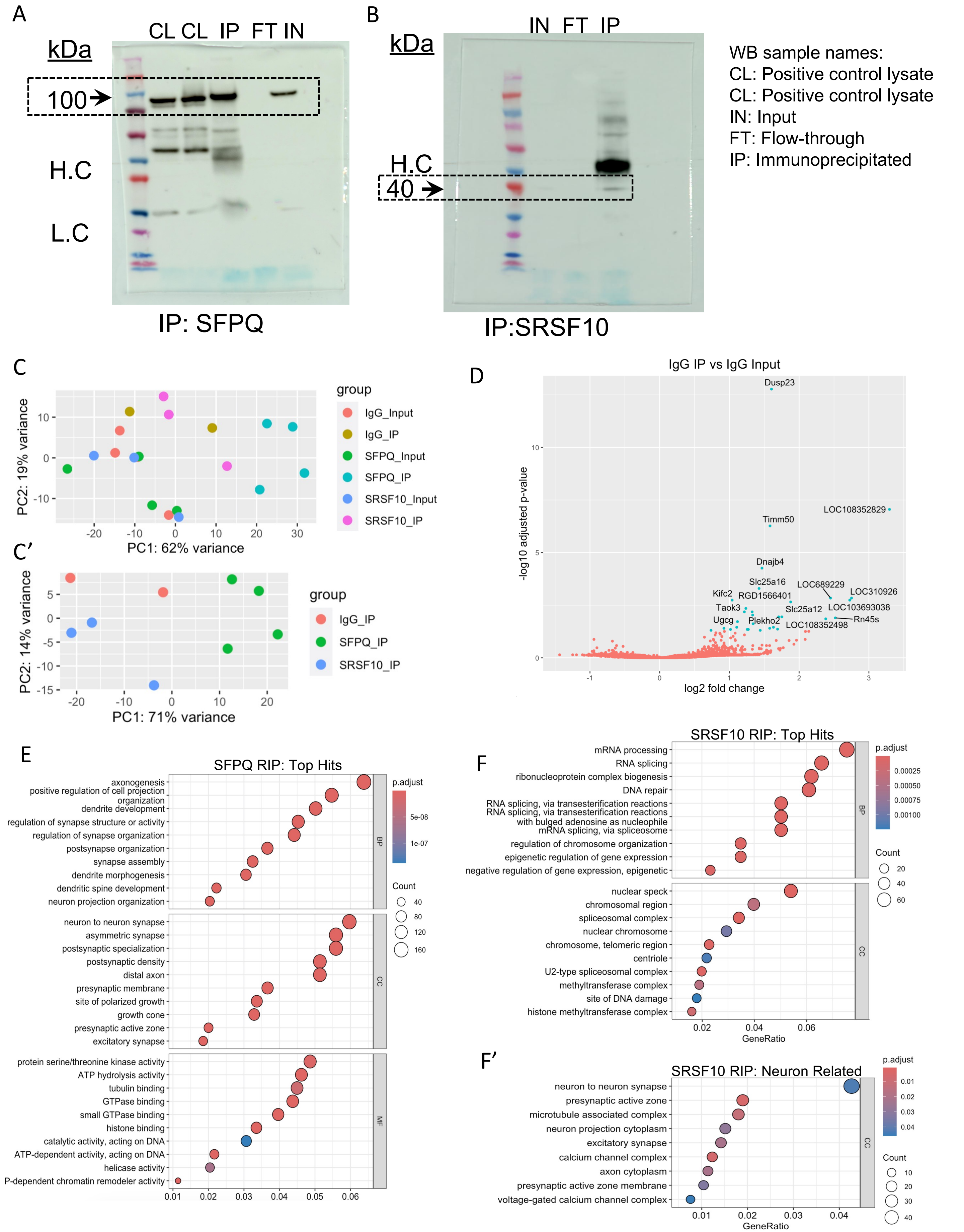
A
CL CL IP FT IN
kDa
100
H.C
L.C
IP: SFPQ
B
IN FT IP
kDa
H.C
40
IP:SRSF10
WB sample names:
CL: Positive control lysate
CL: Positive control lysate
IN: Input
FT: Flow-through
IP: Immunoprecipitated
C
PC2: 19% variance
PC1: 62% variance
group
IgG_Input
IgG_IP
SFPQ_Input
SFPQ_IP
SRSF10_Input
SRSF10_IP
C'
PC2: 14% variance
PC1: 71% variance
group
IgG_IP
SFPQ_IP
SRSF10_IP
D
IgG IP vs IgG Input
-log10 adjusted p-value
log2 fold change
Dusp23
LOC108352829
Timm50
Dnajb4
Slc25a16
LOC689229
Kifc2
RGD1566401
LOC310926
Taok3
Slc25a12
LOC103693038
Ugcg
Plekho2
LOC108352498
Rn45s
E
SFPQ RIP: Top Hits
axonogenesis
positive regulation of cell projection organization
dendrite development
regulation of synapse structure or activity
regulation of synapse organization
postsynapse organization
synapse assembly
dendrite morphogenesis
dendritic spine development
neuron projection organization
neuron to neuron synapse
asymmetric synapse
postsynaptic specialization
postsynaptic density
distal axon
presynaptic membrane
site of polarized growth
growth cone
presynaptic active zone
excitatory synapse
protein serine/threonine kinase activity
ATP hydrolysis activity
tubulin binding
GTPase binding
small GTPase binding
histone binding
catalytic activity, acting on DNA
ATP-dependent activity, acting on DNA
helicase activity
P-dependent chromatin remodeler activity
BP
CC
MF
p.adjust
5e-08
1e-07
Count
40
80
120
160
F
SRSF10 RIP: Top Hits
mRNA processing
RNA splicing
ribonucleoprotein complex biogenesis
DNA repair
RNA splicing, via transesterification reactions
RNA splicing, via transesterification reactions with bulged adenosine as nucleophile
mRNA splicing, via spliceosome
regulation of chromosome organization
epigenetic regulation of gene expression
negative regulation of gene expression, epigenetic
nuclear speck
chromosomal region
spliceosomal complex
nuclear chromosome
chromosome, telomeric region
centriole
U2-type spliceosomal complex
methyltransferase complex
site of DNA damage
histone methyltransferase complex
GeneRatio
p.adjust
0.00025
0.00050
0.00075
0.00100
Count
20
40
60
F'
SRSF10 RIP: Neuron Related
neuron to neuron synapse
presynaptic active zone
microtubule associated complex
neuron projection cytoplasm
excitatory synapse
calcium channel complex
axon cytoplasm
presynaptic active zone membrane
voltage-gated calcium channel complex
GeneRatio
p.adjust
0.01
0.02
0.03
0.04
Count
10
20
30
40

Figure S7

Figure S7. SFPQ and SRSF10 RIP-Sequencing. A, B) Uncropped Western blot images showing SFPQ (A) and SRSF10 (B) protein. C) Principal component analysis demonstrating the variance between SFPQ IP, SRSF10 IP, IgG IP, and paired input samples (n=4 sets/condition). C') Principal component analysis demonstrating the variance between SFPQ IP (n=4 sets), SRSF10 IP (n=3 sets), and IgG IP (n=2 sets) samples with non-zero values only. D) Volcano plots from RIP-Seq experiments demonstrating the mRNA transcripts with significantly higher expression in IgG IP samples as compared to the input fraction from the same DRG neuron cell culture experiments. The most significant differentially expressed transcripts are labeled on the graph. E, F) Gene ontology (GO) enrichment analysis visualized as a dot plot, where each dot represents the most significantly enriched biological processes (BP), cellular compartments (CC), or molecular functions (MF) from the SFPQ IP (E) and SRSF10 IP (F) datasets ($p.adj \leq 0.05$). F') Dot plot demonstrating significantly enriched, neuron-related GO cellular compartments from the SRSF10 IP dataset ($p.adj \leq 0.05$).
