# Supplementary material for: RBP-Driven RNA sorting and local translation establish the molecular identities of individual sensory axons
Source: Nat Commun. 2026 Aug 31;17:10345. doi: 10.1038/s41467-026-77192-x (PMC13623950; doi:10.1038/s41467-026-77192-x)
Supplement: Supplementary file 2 [file 41467_2026_77192_MOESM2_ESM.pdf]

Description of Additional Supplementary Files

File Name: Supplementary Data 1

Description: Normalized Counts for GFP+ Samples

File Name: Supplementary Data 2

Description: Differential Gene Expression Analysis between Central and Peripheral Axons

File Name: Supplementary Data 3

Description: Enriched GO terms: Shared Between Central and Peripheral Axons

File Name: Supplementary Data 4

Description: Differential Gene Expression Analysis between Central Axons and DRG Soma

File Name: Supplementary Data 5

Description: Differential Gene Expression Analysis between Peripheral Axons and DRG soma

File Name: Supplementary Data 6

Description: Enriched GO terms: Central Axons (CA vs. PA)

File Name: Supplementary Data 7

Description: Enriched GO terms: Peripheral Axons (PA vs. CA)

File Name: Supplementary Data 8

Description: Differential Gene Expression Analysis between Pain-seq and TRAP-seq

File Name: Supplementary Data 9

Description: Background Filtered Gene Lists (Higher Confidence)

File Name: Supplementary Data 10

Description: Validated Locally Translated mRNAs in Central and Peripheral Axons

File Name: Supplementary Data 11

Description: Normalized Counts for all RIP Samples

File Name: Supplementary Data 12

Description: Differentially Expressed Genes in SFPQ-IP samples

File Name: Supplementary Data 13

Description: Differentially Expressed Genes in SRSF-IP samples

File Name: Supplementary Data 14

Description: Enriched GO terms: SFPQ IP

File Name: Supplementary Data 15

Description: Enriched GO terms: SRSF IP
