# Supplementary material for: RBP-Driven RNA sorting and local translation establish the molecular identities of individual sensory axons
Source: Nat Commun. 2026 Aug 31;17:10345. doi: 10.1038/s41467-026-77192-x (PMC13623950; doi:10.1038/s41467-026-77192-x)
Supplement: Supplementary file 3 [file 41467_2026_77192_MOESM4_ESM.pdf]

nature portfolio

Corresponding author(s): Rosalind Segal

Last updated by author(s): Jul 19, 2026

Reporting Summary

Nature Portfolio wishes to improve the reproducibility of the work that we publish. This form provides structure for consistency and transparency in reporting. For further information on Nature Portfolio policies, see our Editorial Policies and the Editorial Policy Checklist.

Statistics

For all statistical analyses, confirm that the following items are present in the figure legend, table legend, main text, or Methods section.

n/a	Confirmed	
☐	☒	The exact sample size (*n*) for each experimental group/condition, given as a discrete number and unit of measurement
☐	☒	A statement on whether measurements were taken from distinct samples or whether the same sample was measured repeatedly
☐	☒	The statistical test(s) used AND whether they are one- or two-sided<br>*Only common tests should be described solely by name; describe more complex techniques in the Methods section.*
☐	☒	A description of all covariates tested
☐	☒	A description of any assumptions or corrections, such as tests of normality and adjustment for multiple comparisons
☐	☒	A full description of the statistical parameters including central tendency (e.g. means) or other basic estimates (e.g. regression coefficient) AND variation (e.g. standard deviation) or associated estimates of uncertainty (e.g. confidence intervals)
☐	☒	For null hypothesis testing, the test statistic (e.g. *F*, *t*, *r*) with confidence intervals, effect sizes, degrees of freedom and *P* value noted<br>*Give P values as exact values whenever suitable.*
☒	☐	For Bayesian analysis, information on the choice of priors and Markov chain Monte Carlo settings
☒	☐	For hierarchical and complex designs, identification of the appropriate level for tests and full reporting of outcomes
☐	☒	Estimates of effect sizes (e.g. Cohen's *d*, Pearson's *r*), indicating how they were calculated

Our web collection on statistics for biologists contains articles on many of the points above.

Software and code

Policy information about availability of computer code

Data collection	Sequences for TRAP-seq and RIP-seq were aligned to mouse and rat genomes, respectively, with STAR_2.7.2b. RBP bioinformatic data collection was performed using the oRNAment (23), RBPmap (43), and RPS.renlab (44) online databases. All fluorescent images were collected in a Nikon Eclipse Ni C2 Si laser scanning confocal using NIS Elements Software v4.20.03
Data analysis	RNA-seq analysis of TRAP data, RIP data, and cross-dataset integration was performed in R v4.3.2 for differential gene expression testing f or pairwise, group comparisons (DESeq2) (27) and gene ontological functional annotation (enrichGO) (28,29). Fluorescent micrographs for RNAscope, PLA, and IHC assays were analyzed in Fiji (ImageJ) v2.14.0 using particle analysis. All statistical analyses were performed and graphs plotted in either R v4.3.2 or GraphPad Prism v10. Schematics were created using BioRender (acknowledged).<br><br>• Novel code used to generate the web resource has been deposited to GitHub (http://github.com/Renthal-Lab/CompartmentTRAPShiny): DOI: 10.5281/zenodo.21176607

For manuscripts utilizing custom algorithms or software that are central to the research but not yet described in published literature, software must be made available to editors and reviewers. We strongly encourage code deposition in a community repository (e.g. GitHub). See the Nature Portfolio guidelines for submitting code & software for further information.

Data

Policy information about availability of data

All manuscripts must include a data availability statement. This statement should provide the following information, where applicable:

- Accession codes, unique identifiers, or web links for publicly available datasets
- A description of any restrictions on data availability
- For clinical datasets or third party data, please ensure that the statement adheres to our policy

• All raw and processed TRAP-seq and RIP-seq data from this study were deposited to Gene Expression Omnibus at GEO:GSE289550 and GEO:GSE289551 and are publicly available: https://www.ncbi.nlm.nih.gov/geo/query/acc.cgi?acc=GSE289550
https://www.ncbi.nlm.nih.gov/geo/query/acc.cgi?acc=GSE289551
• TRAP-seq data are also available for browsing and analysis via the Pain-seq multi-omic data resource at http://painseq.shinyapps.io/CompartmentTRAP/.
The TRAP-seq, RIP-seq, and integrative cross dataset analyses performed in this study are available in the Supplementary Data files.
• All visual data analyzed in this study can be found in the Source Data file.
• Requests for further information and resources should be directed to the lead contact, Rosalind Segal (Rosalind_segal@dfci.harvard.edu).

Research involving human participants, their data, or biological material

Policy information about studies with human participants or human data. See also policy information about sex, gender (identity/presentation), and sexual orientation and race, ethnicity and racism.

Reporting on sex and gender	*Use the terms sex (biological attribute) and gender (shaped by social and cultural circumstances) carefully in order to avoid confusing both terms. Indicate if findings apply to only one sex or gender; describe whether sex and gender were considered in study design; whether sex and/or gender was determined based on self-reporting or assigned and methods used.<br>Provide in the source data disaggregated sex and gender data, where this information has been collected, and if consent has been obtained for sharing of individual-level data; provide overall numbers in this Reporting Summary. Please state if this information has not been collected.<br>Report sex- and gender-based analyses where performed, justify reasons for lack of sex- and gender-based analysis.*
Reporting on race, ethnicity, or other socially relevant groupings	*Please specify the socially constructed or socially relevant categorization variable(s) used in your manuscript and explain why they were used. Please note that such variables should not be used as proxies for other socially constructed/relevant variables (for example, race or ethnicity should not be used as a proxy for socioeconomic status).<br>Provide clear definitions of the relevant terms used, how they were provided (by the participants/respondents, the researchers, or third parties), and the method(s) used to classify people into the different categories (e.g. self-report, census or administrative data, social media data, etc.)<br>Please provide details about how you controlled for confounding variables in your analyses.*
Population characteristics	*Describe the covariate-relevant population characteristics of the human research participants (e.g. age, genotypic information, past and current diagnosis and treatment categories). If you filled out the behavioural & social sciences study design questions and have nothing to add here, write "See above."*
Recruitment	*Describe how participants were recruited. Outline any potential self-selection bias or other biases that may be present and how these are likely to impact results.*
Ethics oversight	*Identify the organization(s) that approved the study protocol.*

Note that full information on the approval of the study protocol must also be provided in the manuscript.

Field-specific reporting

Please select the one below that is the best fit for your research. If you are not sure, read the appropriate sections before making your selection.

☒ Life sciences ☐ Behavioural & social sciences ☐ Ecological, evolutionary & environmental sciences

For a reference copy of the document with all sections, see nature.com/documents/nr-reporting-summary-flat.pdf

Life sciences study design

All studies must disclose on these points even when the disclosure is negative.

Sample size	Sample sizes for RNA-sequencing experiments were determined a priori based on estimates from previously published datasets in similar tissue types and experimental conditions. Sample size for in vivo imaging studies were determined based on prior in vivo studies examining gene expression in sciatic nerves and dorsal roots, including our own pilot data. Furthermore, power calculations were used to determine the number of animals (n=4) required to detect a ≥25% change in the proportion of positive signal with a standard deviation of ~10%, an alpha of 0.05, and 80% power. All attempts to replicate experiments were successful.

Data exclusions	No data were excluded from the study.
Replication	To ensure reproducibility of all studies, all experiments were conducted on at least four independent biological cohorts and processed independently. All experiments were run with proper negative controls, such as no primary antibodies, or negative control probes, where applicable. Furthermore, tissue processing and experimental staining and hybridization conditions were rigorously controlled.
Randomization	Sciatic nerves and dorsal roots of all mice were analyzed for sequencing, RNAscope, PLA, and IHC assays. Samples were randomly assigned to assess expression of select targets.
Blinding	Blinding was not possible for this study as landmarks could be used to distinguish the sciatic nerves from dorsal roots.

Reporting for specific materials, systems and methods

We require information from authors about some types of materials, experimental systems and methods used in many studies. Here, indicate whether each material, system or method listed is relevant to your study. If you are not sure if a list item applies to your research, read the appropriate section before selecting a response.

Materials & experimental systems

n/a	Involved in the study
	[x] Antibodies
[x]	Eukaryotic cell lines
[x]	Palaeontology and archaeology
	[x] Animals and other organisms
[x]	Clinical data
[x]	Dual use research of concern
[x]	Plants

Methods

n/a	Involved in the study
[x]	ChIP-seq
[x]	Flow cytometry
[x]	MRI-based neuroimaging

Antibodies

Antibodies used

Mouse anti-GFP-19C8 Memorial Sloan-Kettering Monoclonal Antibody Facility Cat#Htz-GFP-19C8; RRID:AB_2716737 (2 μg per mouse for TRAP)
Mouse anti-GFP-19F7 Memorial Sloan-Kettering Monoclonal Antibody Facility Cat#Htz-GFP-19F7; RRID:AB_2716736 (2 μg per mouse for TRAP)
Mouse anti-Puromycin Millipore Sigma Cat#MABE343; RRID:AB_2566826 (20 μg for Duolink Probemarker kit; 1:250 conjugated for PLA)
Mouse anti-Puromycin-647 Millipore Sigma Cat#MABE343-AF647; RRID:AB_2736876 (1:1000 for fIHC)
Mouse anti-TUBB3/TUJ1-488 BioLegend Cat#803203; RRID:AB_2564757 (1:500 for fIHC)
Rabbit anti-5HTR3A Abcam Cat#AB38148; RRID:AB_945424 (1:500 for PLA)
Rabbit anti-SCN11A Alomone Labs Cat# ASC-017; RRID:AB_2040200 (1:500 for PLA)
Rabbit anti-SCN2A Alomone Labs Cat# ASC-002; RRID:AB_2040005 (1:500 for PLA)
Rabbit anti-HCN1 Alomone Labs Cat# APC-056; RRID:AB_2039900 (1:500 for PLA)
Rabbit anti-SFPQ Abcam Cat#ab38148; RRID:AB_945424 (1:500 IHC; 1:1000 WB, 5μg for IP)
Rabbit anti-HuR Proteintech Cat#11910-1-AP; RRID:AB_11182183 (1:250 for fIHC)
Rabbit anti-SRSF10 Bioss #bs-13229R (1:400 fIHC; 1:1000 WB; 5μg for IP)
Rabbit anti-RFP Rockland Cat#RL600-401-379 (1:500 for fIHC)
Donkey anti-rabbit (H+L), Alexa Fluor 568 ThermoFisher Scientific Cat#A10042; RRID:AB_2556622 (1:1000 for fIHC)
Goat anti-rabbit IgG (H+L), HRP conjugate Bio-Rad Cat#1706515; RRID:AB_11125143 (1:10,000 for WB)

Validation

GFP antibodies were validated by Myriam Heiman using EGFP-L10a and empty vectors expressed in HEK293T cells (see reference #27)

anti-Puromycin antibodies (Millipore Sigma, Cat#MABE343) were validated with cycloheximide treatment per the manufacturer website: https://www.sigmaaldrich.com/US/en/product/mm/mabe343?srsltid=AfmBOooh87F0zI9ENHXP7mzAyScSirwF0ixovY4Eu8S1CPmEvfNcW2Vt

anti-TUBB3/TUJ1-488 antibody (BioLegend Cat#803203) was validated by the manufacturer for IHC in neuronal cells: https://www.biolegend.com/de-de/products/alexa-fluor-488-anti-tubulin-beta-3-tubb3-antibody-10828

anti-5HTR3A antibody (Abcam #AB38148) was validated by the Segal lab on Htr3a cKO tissue (unpublished) and by the manufacturer, using WB of tissue protein: https://www.abcam.com/en-us/products/primary-antibodies/5ht3a-receptor-5ht3-antibody-epr24323-75-ab271031.

anti-SCN11A antibody (Alomone Labs Cat# ASC-017) was validated with blocking peptide by the manufacturer: https://www.alomone.com/products/asc-017-anti-scn11a-na-sub-v-sub-1-9-antibody?srsltid=AfmBOorVVPDaM-ei7QEQr3ZceEr3493RAbpx-TkHnREiqHQ7DGqa52kj

anti-SCN2A antibody (Alomone Labs Cat# ASC-002) was knock-out validated by the manufacturer: https://www.alomone.com/products/asc-002-anti-scn2a-na-sub-v-sub-1-2-antibody

anti-HCN1 antibody (Alomone Labs Cat# APC-056) was knock-out validated by the manufacturer: https://www.alomone.com/products/apc-056-anti-hcn1-antibody?srsltid=AfmBOopug8zUwpWnotKdR2WmxmLBVWd6diq-KWnGAk_vFmYmdW7Iyqnz

anti-HUR antibody (Proteintech #11910-1-AP) was validated by the manufacturer by WB, IP, and KD/KO-validated in 32 publications as noted by the manufacturer website: https://www.ptglab.com/products/HuR-Antibody-11910-1-AP.htm?srsltid=AfmBOoooWhlRCuUylPgWJMUgm79GLaUWTstnqzOY_Ct2jqIBeRWHX6sr#publications. Primary only (no secondary) controls were performed in tandem with fIHC assays.

anti-SRSF10 antibody (Bioss; #bs-13229R) was validated by WB in ovary tissue lysate by the manufacturer: https://www.thermofisher.com/antibody/product/FUSIP1-Antibody-Polyclonal/BS-13229R. Primary only (no secondary) controls were performed in tandem with fIHC assays.

anti-SFPQ antibody (Abcam Cat#ab38148) and anti-SRSF10 (MBL; #RN064PW) were IP-WB validated in Figure S7 of the manuscript. anti-SFPQ (Abcam) was also validated for WB, IHC, and ICC by the manufacturer: https://www.abcam.com/en-us/products/primary-antibodies/sfpq-antibody-ab38148. Primary only (no secondary) controls were performed in tandem with fIHC assays.
anti-SRSF10 antibody (MBL) was validated for WB by the manufacturer: https://www.mblbio.com/bio/g/dtl/A/index.html?pcd=RN064PW.

anti-RFP (Rockland Cat#RL600-401) was validated in our lab by assessing specificity for labeling Tdtomato expressing axons in Figures 5-7 of the manuscript.

Animals and other research organisms

Policy information about studies involving animals; ARRIVE guidelines recommended for reporting animal research, and Sex and Gender in Research

Laboratory animals	Mouse Strains: L10a-eGFPfl/fl (B6;129S4-Gt(ROSA)26Sortm9(EGFP/Rpl10a)Amc/J) mice were the generous gift of Myriam Heiman (Massachusetts Institute of Technology)(24). Nav1.8cre (B6.129-Scn10atm2(cre)Jwo/H) and TdTomatofl/fl (B6.Cg-Gt(ROSA)26Sortm14(CAG-tdTomato)Hze/J) mice were kindly provided by David Ginty (Harvard Medical School).<br>For in vivo TRAP studies, Nav1.8cre/+ and L10a-eGFPfl/fl mice were crossed to generate Nav1.8cre;L10a-eGFPfl/fl mice (GFP+) and L10a-eGFPfl/fl (GFP-) littermate controls. For tissue collection and histology, Nav1.8cre and TdTomatofl/fl mice were crossed to generate Nav1.8cre;TdTomatofl/fl mice with labelled sensory neurons.<br><br>Nav1.8-Cre;L10a-eGFP mice aged P3-7 and L10a-eGP littermate controls were used for all TRAP-seq studies.<br>Nav1.8cre;TdTomatofl/fl mice aged P3-7 were used for Puro-PLA, RNAscope, and fluorescent IHC studies .<br>Nav1.8cre;TdTomatofl/fl mice mice aged 2mos were also used for Puro-PLA studies.<br><br>Timed pregnant Sprague-Dawley rats were purchased from Charles River for primary DRG neuron isolation at E15. Embryos were used for RIP-seq experiments.
Wild animals	*Provide details on animals observed in or captured in the field; report species and age where possible. Describe how animals were caught and transported and what happened to captive animals after the study (if killed, explain why and describe method; if released, say where and when) OR state that the study did not involve wild animals.*
Reporting on sex	Due to constraints in the TRAP protocol, we were limited to pooling both male and female mice at postnatal week 1 to generate sufficient material for TRAP sequencing. Therefore, we also pooled sex for the adults (n=4 males, n=3 females). While pooling across sexes increased the sample size, it also introduced the potential for masking or confounding sex-based biological differences in gene expression. Future studies that separate male and female mice or incorporate sex as a biological variable could provide more detailed insights into sex-specific differences in axons.
Field-collected samples	*For laboratory work with field-collected samples, describe all relevant parameters such as housing, maintenance, temperature, photoperiod and end-of-experiment protocol OR state that the study did not involve samples collected from the field.*
Ethics oversight	All experimental procedures were conducted in accordance with the National Institutes of Health (NIH) guidelines and were approved by the Dana-Farber Cancer Institute (DFCI) Institutional Animal Care and Use Committee (IACUC) in protocol #01-133.

Note that full information on the approval of the study protocol must also be provided in the manuscript.

Plants

Seed stocks

Report on the source of all seed stocks or other plant material used. If applicable, state the seed stock centre and catalogue number. If plant specimens were collected from the field, describe the collection location, date and sampling procedures.

Novel plant genotypes

Describe the methods by which all novel plant genotypes were produced. This includes those generated by transgenic approaches, gene editing, chemical/radiation-based mutagenesis and hybridization. For transgenic lines, describe the transformation method, the number of independent lines analyzed and the generation upon which experiments were performed. For gene-edited lines, describe the editor used, the endogenous sequence targeted for editing, the targeting guide RNA sequence (if applicable) and how the editor was applied.

Authentication

Describe any authentication procedures for each seed stock used or novel genotype generated. Describe any experiments used to assess the effect of a mutation and, where applicable, how potential secondary effects (e.g. second site T-DNA insertions, mosiacism, off-target gene editing) were examined.
