# Supplementary material for: RBP-Driven RNA sorting and local translation establish the molecular identities of individual sensory axons
Source: Nat Commun. 2026 Aug 31;17:10345. doi: 10.1038/s41467-026-77192-x (PMC13623950; doi:10.1038/s41467-026-77192-x)
Supplement: Supplementary file 4 [file 41467_2026_77192_MOESM5_ESM.pdf]

nature portfolio

Peer Review File

RBP-Driven RNA Sorting and Local Translation Establish the Molecular Identities of Individual Sensory Axons

Corresponding Author: Dr Rosalind Segal

This file contains all reviewer reports in order by version, followed by all author rebuttals in order by version.

Version 0:

Reviewer comments:

Reviewer #1

(Remarks to the Author)
The molecular differences between the peripheral and central axonal branches of DRG sensory neurons have been a long-standing question. The study by Silagi et al. aims to tackle this difficult problem by examining the local translatome of the two axonal branches of nociceptive neurons. Overall, the study provides strong evidence that the axonal branches indeed differ in their translatome, presumably through the action of different RBPs. The generated data will be a highly valuable resource for groups working on sensory neurons biology and pathology, as well as for groups that work on the general mechanisms of local translation.
I have some comments and suggestions, as outlined below.
Fig. 1G: Bcl2l2 does not show a statistically significant difference between the neuronal compartments. Why do the authors state that it is enriched in the PA compartment?
Fig. 4: For 5HTR3A, most of the signal is not in the Na1.8 axons. How do the authors interpret this? Is this background or signal from other axons? Is this gene enriched in nociceptive neurons or in other neuronal types? There is a large amount of available single-cell data that could address this question.
Fig. 6: The authors examine 3′ UTRs in search of RBP binding sites. From which database were the 3′ UTR sequences obtained—were they generated by the authors in this study or taken from RefSeq? This point is highly important, as it has been demonstrated for several genes that axonally localized transcripts have differential 3′ UTRs.
The authors use two sets of data: the first is based on fold change and p-value, and in the second they filter out many genes based on background expression of non-neuronal genes. They then continue the validation studies only with this filtered set. Based on this, I wonder what the added value of the first data set is.

Reviewer #2

(Remarks to the Author)
This paper by Silagi et al addresses how axon-branch–specific gene expression is regulated by local translation. The choice of dorsal root ganglion (DRG) neurons as a model system is excellent, as the peripheral and central axons of DRG neurons serve physiologically distinct roles as signal detectors and transmitters, respectively.
The authors employ TRAP-seq of Nav1.8-Cre–labeled neurons as a central approach, defining L1–L6 DRGs as the soma compartment, sciatic nerve as the peripheral axon (PA), and L1–L6 spinal cord/dorsal root tissue as the central axon (CA). Using this strategy, they generate two datasets: a more inclusive (“generous”) dataset and a more stringent dataset obtained by subtraction against Cre-negative controls.

Using the generous dataset, the authors conclude that PA and CA possess distinct translatomes and integrate these results with single-cell RNA-seq data from neuropathic pain models (Pain-seq) to interpret compartment-specific changes in disease states. Validation experiments—including puromycin-PLA for nascent protein detection and RIP-seq of SFPQ and SRSF10—primarily focus on targets drawn from the more stringent dataset.

The conclusion that SFPQ and SRSF10 regulate peripheral- and central-axon–biased mRNA transport and translation is highly significant, with broad implications for neuronal cell biology, RNA granule biochemistry, mRNA selectivity, and the molecular mechanisms of pain. The datasets provided in this study will be valuable resources for the field.

I have the following comments on the manuscript that the authors might consider to make their manuscript even stronger.

1. Choice of dataset for main conclusions
The authors rely primarily on the generous dataset to support the conclusion that CA and PA harbor distinct translatomes (Figs. 1–2). As the authors acknowledge, TRAP-seq data inevitably include substantial background RNA from surrounding tissues, which in this case differ between CA (spinal cord), PA (sciatic nerve), and soma (DRG).
In contrast, the more stringent dataset—defined by differential enrichment relative to matched Cre-negative controls—is presented only in supplementary figures (Fig. S3). Because the central conclusions regarding axon-branch specificity can also be supported using this higher-confidence dataset, it would strengthen the manuscript to promote Fig. S3 to the main figures and move Figs. 1–2 to the supplement.

2. Interpretation of the generous dataset
In several instances, functional interpretations of the generous dataset appear to exceed what can be confidently inferred from the data. For example, the statement that "DRG central axons also have the capacity to remodel postsynaptic machinery" is difficult to support given that some CA-enriched transcripts (eg ChAT, Gad2) are not normally expressed by DRG neurons but are characteristic of spinal cord neurons. Similarly, PA-enriched transcripts include Cacna1s, a muscle-specific calcium channel not normally expressed by DRG neurons, raising the possibility of tissue contamination in sciatic nerve preparations. These observations suggest that conclusions drawn from the generous dataset should be more cautiously framed (for example, when comparisons involve tissues with shared background composition) or explicitly limited to the stringent dataset.

3. Mechanism of SFPQ asymmetry
The finding that SFPQ is selectively enriched in peripheral axons is highly intriguing. Given its central role in the authors' model, it would strengthen the Discussion to elaborate on potential mechanisms governing the asymmetric localization of SFPQ itself.

Reviewer #3

(Remarks to the Author)
The authors used a TRAP-seq approach with Nav1.8-Cre x L10aGFPfl/fl mice to identify mRNAs translated in peripherally vs. centrally projecting DRG axons in 1 week old mice. Surprisingly, there are different RNA populations in the peripheral vs. central axons of these unipolar neurons. They identify the RNA binding proteins SFPQ and SRSF10 as enriched in peripheral vs. central axons based on motif enrichment across the translatome datasets and use IP to identify enriched binding for mRNA regulons by these proteins. Overall, the study is very well-done and provides a key scientific advance for understanding how molecular identities of subcellular regions/domains can be crafted by RNA transport and local translation. Ribosome profiling used here is a strength of the study since it gets past just the presence of the RNA and shows translation or at least ribosome interaction of the mRNAs. The authors provide validation of compartment selective translation for some identified mRNAs using puro-PLA type experiments. I only have a few comments, most of which can be settled with clarification in the text or modifications.

Major points:

1. It is puzzling that the authors use 1 week old mice and infer physiological functions that we typically do not consider at that age – i.e., pain and neuropathy as they cross reference with paclitaxel transcriptomics work. I suspect there were technical reasons for using young mice DRGs, but I think the authors should make that clear to the readers and clarify whether there would be developmental differences expected.

2. Considering # 1, is the cross-dataset comparison between this study's TRAP dataset and the single-cell RNA-Seq dataset of DRG neurons treated with paclitaxel a fair comparison since adult mice and only somas (i.e., not central or peripheral axons) were used for the paclitaxel study? The authors need to point out this limitation of the study and the complications that my result from that. I think they also need to back down statements like in the summary where they state encoded proteins 'confer distinct electrophysiological, synaptic, and regenerative properties' – statements in the discussion are also overreaching their data.

3. In supplemental figure 3, several ion channels were identified that were locally translated in peripheral axons. The authors suggest that the local translation of these ion channels may modify pain responses. Is there a way to show this in vivo with knockdown studies?

4. The comparing motifs across the peripherally vs. centrally enriched TRAP-seq data sets is a clever approach to get at potential compartment selective RBPs. The authors refer to these as 'binding sites' later in the text. I think they need to be clear that those are potential or putative binding sites as their pull down data do not address specific sites of binding (unless I missed that detail).

5. For Figure 6, the Nav1.8-tdT images for A look more punctate and not continuous like the Nav1.8-tdT labeled axons in panels B & C. The sciatic nerve panel for Nav1.8-tdT in panel A looks like it is showing Schwann cell nuclei.

Minor points:

1. On line 51, the authors reference Campbell et al (2001) and a recent review from the Sotelo group to emphasize that ribosomes are present in and mRNAs translated within axons. Notably, Zheng et al's publication on ribosomes in DRG axons (J Neurosci 21, 9291) was published a few weeks earlier than the Campbell paper. The authors should reference both of these here.

2. For clarification, how far away from the soma were the sciatic nerves and dorsal roots collected? Should considerations be made for this distance since there may be differences between translation at the distal ends versus closer to the soma?

3. For line 344-6, sentence indicating that SFPQ IF is consistent with their prediction for enrichment in peripheral axons – I thought the senior author's lab had previously published selective peripheral localization of SFPQ from their Bclw work? If so, they should reference that here or somewhere in this section.

4. For Figures 5 and 6, the authors need to indicate in the figure that the merged images include DAPI.

Version 1:

Reviewer comments:

Reviewer #1

(Remarks to the Author)
All my comments are addressed in the revised manuscript

Reviewer #2

(Remarks to the Author)
The revised manuscript presents all key datasets as main figures, which are arranged in a way that makes it easier to follow the flow of ideas leading to the main conclusion of this study that DRG neurons have a mechanism that enables axon branch-specific mRNA transport. The datasets are presented in a way that acknowledges key limitations of the technology (i.e., the background of immunoprecipitation), which are rigorously monitored by matching experimental controls. The discussion is balanced and cautious. This manuscript tackles an important question in neuronal cell biology with a cutting-edge technique, and the conclusions and datasets will be of interest to the wide audience of Nature Communications. I support the publication of the revised manuscript.

Reviewer #3

(Remarks to the Author)
The authors have addressed all of my and (I think) the other reviewers' concerns. The addition of adult DRG neuron data strengthens the manuscript and the authors have included limitations of their data that will be important for the reader. I think this manuscript represents an advance for the field and should be an important technical resource. I only have 2 minor comments:

1. Pg 4, line 21 – concluding sentence is missing a period.

2. Pg 3, lines 18-20 – the authors refer to 'higher expression' of axonally translated genes. They should refer to this as higher axonal mRNA levels or axonal translation in the peripheral compartment.

Open Access This Peer Review File is licensed under a Creative Commons Attribution 4.0 International License, which permits use, sharing, adaptation, distribution and reproduction in any medium or format, as long as you give appropriate credit to the original author(s) and the source, provide a link to the Creative Commons license, and indicate if changes were made.
In cases where reviewers are anonymous, credit should be given to 'Anonymous Referee' and the source.
The images or other third party material in this Peer Review File are included in the article's Creative Commons license, unless indicated otherwise in a credit line to the material. If material is not included in the article's Creative Commons license and your intended use is not permitted by statutory regulation or exceeds the permitted use, you will need to obtain permission directly from the copyright holder.
To view a copy of this license, visit https://creativecommons.org/licenses/by/4.0/

Authors Response to Reviewer Comments

We were very pleased with the positive comments from the reviewers, and we found the critiques to be extremely helpful in improving the study. We have carried out additional experiments and made textual changes to address all the issues raised. The major changes that we made in this revised manuscript, include moving the former Supplemental Figure S3 into the new main Figure 4, as suggested by the reviewers. We have carried out additional experiments suggested by reviewer 3 to determine whether the differences in the translatomes of the central and peripheral axons are maintained in adulthood, and are modulated by an insult such as chemotherapy. These exciting new experiments are in the new Figure 6 and the new Supplemental Figure S4. Finally, as suggested by the reviewers we have improved our statistical analysis throughout the manuscript and included a source data file for all bar graphs and dot plots. We think that the revised paper will be of broad interest to neuroscientists, including those studying axon degeneration, cancer neuroscience, and calcium regulation, and we hope that this will be deemed appropriate for publication in *Nature Communications*. In the manuscript file, revisions are in red, and we highlight in yellow text what directly addresses individual comments from the reviewers. Our detailed responses to the reviewers are included below, with our responses in red.

Reviewer #1 (Remarks to the Author):

The molecular differences between the peripheral and central axonal branches of DRG sensory neurons have been a long-standing question. The study by Silagi et al. aims to tackle this difficult problem by examining the local translatome of the two axonal branches of nociceptive neurons. Overall, the study provides strong evidence that the axonal branches indeed differ in their translatome, presumably through the action of different RBPs. The generated data will be a highly valuable resource for groups working on sensory neurons biology and pathology, as well as for groups that work on the general mechanisms of local translation.

I have some comments and suggestions, as outlined below.

We thank the reviewer for these positive comments about the study. We appreciate the questions and suggestions which we have addressed as below. We believe these changes greatly improve our study.

Fig. 1G: Bcl2l2 does not show a statistically significant difference between the neuronal compartments. Why do the authors state that it is enriched in the PA compartment?

We agree that the original one-way ANOVA analysis with Tukey's correction across DRG, PA, and CA did not identify a significant pairwise difference in bcl2l2 (Bclw), and our wording was therefore too strong. Our primary biological question in this panel is the relative abundance of Bclw between axonal compartments (PA vs CA); DRG was included for context because it was available in the dataset.

We have revised Fig. 1G and the accompanying text/legend to reflect an a priori PA–CA comparison using an unpaired t-test of DESeq2-normalized counts for all genes. We now report the PA vs CA log2 fold change and corresponding p-value. We continue to show DRG values for reference, but these are not used in this statistical comparison. The text has been updated to state that Bclw is higher in PA than CA, and we avoid the term "enriched".

Fig. 4: For 5HTR3A, most of the signal is not in the Nav1.8 axons. How do the authors interpret this? Is this background or signal from other axons? Is this gene enriched in nociceptive neurons or in other neuronal types? There is a large amount of available single-cell data that could address this question.

We agree that 5HTR3A signal is not restricted to Nav1.8+ axons in our P3 tissue. To better interpret this, we examined publicly available developmental single-cell RNA-seq data (Sharma & Ginty, 2020). These data indicate that Htr3a expression is enriched in embryonic and early postnatal neuronal populations and is not tightly restricted to Scn10a+ nociceptors at these stages.

Given that our experiments were performed at ~P3, a period of ongoing sensory neuron maturation, the broader 5HTR3A signal we observe likely reflects developmental expression patterns rather than background staining. We have revised the text to clarify that 5HTR3A is not exclusively nociceptor-specific at this developmental stage, saying on pg.5; line 56 – pg. 6; line 3 "Consistent with developmental single-cell datasets from Sharma *et al.* showing that *Htr3a* expression at early postnatal stages is expressed in a broad population of DRG neurons [26], HTR3A is translated in both Nav1.8 positive and negative central axons."

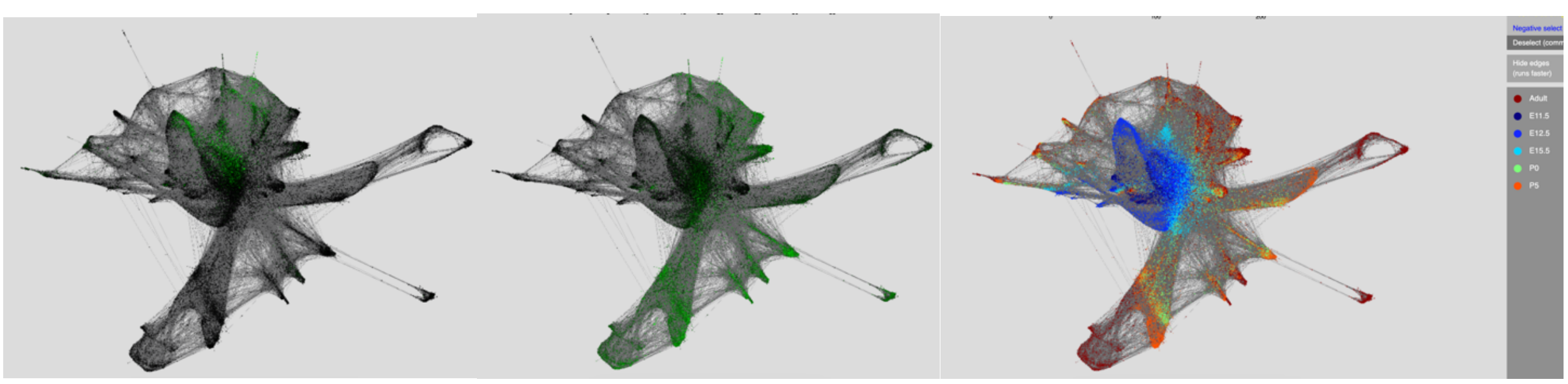

Htr3a Scn10a Timepoint Clusters

In addition, Htr3a translation is not restricted to neurons. Puro-PLA in sciatic nerve revealed translation in perineural cells (see below; yellow dashed boxes). In the text we now say on pg. 6; lines 3-4, "HTR3A is also translated in perineural cells at these timepoints, as we observed in sciatic nerve tissue."

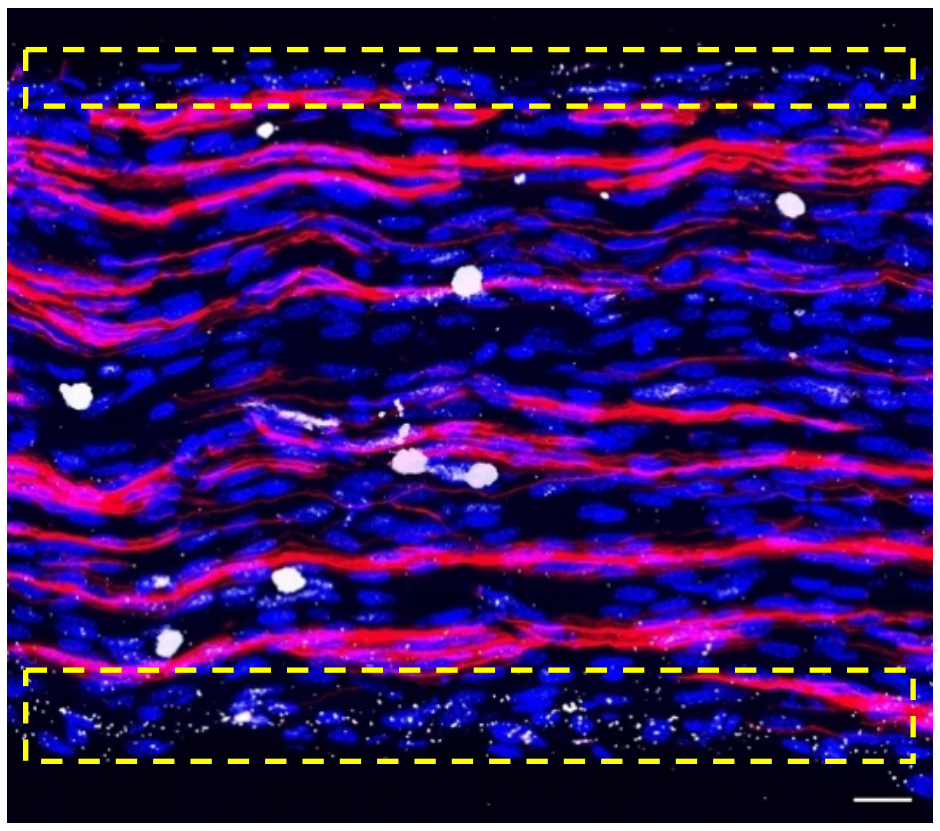

Fig. 6: The authors examine 3′ UTRs in search of RBP binding sites. From which database were the 3′ UTR sequences obtained—were they generated by the authors in this study or taken from RefSeq? This point is highly important, as it has been demonstrated for several genes that axonally localized transcripts have differential 3′ UTRs.

We thank the reviewer for raising this important point. The 3′ UTR sequences used for RBP motif analysis were obtained from the UCSC Genome Browser (GRCm39/mm39 assembly). For each gene in our locally translated transcript list, we extracted the longest annotated 3′ UTR corresponding to the UCSC RefSeq transcript model. These sequences were reference-annotated and were not generated de novo in this study. We agree that alternative polyadenylation and differential 3′ UTR usage can influence axonal localization. Our current analysis therefore reflects motif enrichment within reference-annotated 3′ UTRs and does not distinguish alternative isoforms. We have clarified this in the Methods section on pg. 16; lines 1-7.

The authors use two sets of data: the first is based on fold change and p-value, and in the second they filter out many genes based on background expression of non-neuronal genes. They then continue the validation studies only with this filtered set. Based on this, I wonder what the added value of the first data set is.

We thank the reviewer for this important question. The two datasets were designed to address complementary analytical goals and differ in sensitivity versus specificity. The initial dataset defines the compartment-resolved translatomes and was used for principal component analysis, GO enrichment, and cross-dataset integration. This dataset is biologically inclusive and statistically powered to identify compartment-defined translational programs. Next, we subsequently applied a stringent background filter using GFP-negative controls to generate a specificity-enriched subset for targeted validation experiments. While this analysis increases confidence in Nav1.8+ DRG neuron derivation, it is intentionally conservative and may risk false negatives. Notably, several well-established axonally translated mRNAs were retained in the primary dataset but removed by the background filter, consistent with their broader expression across cell types. Therefore, the full dataset more comprehensively represents compartment-specific translational programs, meanwhile the filtered dataset is appropriate for high-confidence validation studies. We have clarified this distinction in the revised manuscript on page. 5; lines 22-26, saying "While this additional analysis increases confidence that the selected components are translated in axons of Nav1.8+ DRG neurons, it is intentionally conservative. Accordingly, global analyses of compartment-specific translational programs were performed on the full dataset as discussed above, whereas the filtered dataset defines high-confidence Nav1.8-specific transcripts used for downstream validation."

Reviewer #2 (Remarks to the Author):

This paper by Silagi et al addresses how axon-branch–specific gene expression is regulated by local translation. The choice of dorsal root ganglion (DRG) neurons as a model system is excellent, as the peripheral and central axons of DRG neurons serve physiologically distinct roles as signal detectors and transmitters, respectively.
The authors employ TRAP-seq of Nav1.8-Cre–labeled neurons as a central approach, defining L1–L6 DRGs as the soma compartment, sciatic nerve as the peripheral axon (PA), and L1–L6 spinal cord/dorsal root tissue as the central axon (CA). Using this strategy, they generate two datasets: a more inclusive ("generous") dataset and a more stringent dataset obtained by subtraction against Cre-negative controls.

Using the generous dataset, the authors conclude that PA and CA possess distinct translatomes and integrate these results with single-cell RNA-seq data from neuropathic pain models (Pain-seq) to interpret compartment-specific changes in disease states. Validation experiments—including puromycin-PLA for nascent protein detection and RIP-seq of SFPQ and SRSF10—primarily focus on targets drawn from the more stringent dataset.

The conclusion that SFPQ and SRSF10 regulate peripheral- and central-axon–biased mRNA transport and translation is highly significant, with broad implications for neuronal cell biology, RNA granule biochemistry, mRNA selectivity, and the molecular mechanisms of pain. The datasets provided in this study will be valuable resources for the field.

We thank the reviewer for these kind comments.

I have the following comments on the manuscript that the authors might consider to make their manuscript even stronger.

1. Choice of dataset for main conclusions
The authors rely primarily on the generous dataset to support the conclusion that CA and PA harbor distinct translatomes (Figs. 1–2). As the authors acknowledge, TRAP-seq data inevitably include substantial background RNA from surrounding tissues, which in this case differ between CA (spinal cord), PA (sciatic nerve), and soma (DRG).
In contrast, the more stringent dataset—defined by differential enrichment relative to matched Cre-negative controls—is presented only in supplementary figures (Fig. S3). Because the central conclusions regarding axon-branch specificity can also be supported using this higher-confidence dataset, it would strengthen the manuscript to promote Fig. S3 to the main figures and move Figs. 1–2 to the supplement.

We appreciate the reviewer's comments, which are similar to those of the previous reviewer (see Reviewer 1- #4). Therefore, to strengthen the manuscript, we have moved the stringent background-filtered analysis (previously Fig. S3) into the main figures as Fig. 4. This analysis confirms that transcripts enriched in GFP+ samples relative to matched GFP− controls retain the compartment-specific trends observed in Figs. 1–2. Importantly, while this conservative filtering step increases specificity for Nav1.8-derived transcripts, the broader fold-change/padj-defined dataset enhances the scope of the overall compartment-level data. Together, these complementary analyses demonstrate that our conclusions regarding axon-branch–specific translation are robust to background filtering.

2. Interpretation of the generous dataset
In several instances, functional interpretations of the generous dataset appear to exceed what can be confidently inferred from the data. For example, the statement that "DRG central axons also have the capacity to remodel postsynaptic machinery" is difficult to support given that some CA-enriched transcripts (eg ChAT, Gad2) are not normally expressed by DRG neurons but are characteristic of spinal cord neurons. Similarly, PA-enriched transcripts include Cacna1s, a muscle-specific calcium channel not normally expressed by DRG neurons, raising the possibility of tissue contamination in sciatic nerve preparations. These observations suggest that conclusions drawn from the generous dataset should be

more cautiously framed (for example, when comparisons involve tissues with shared background composition) or explicitly limited to the stringent dataset.

We thank the reviewer for this important point. We agree that the inclusive fold-change/padj-defined dataset includes transcripts that may derive from the surrounding tissue composition of each compartment (e.g., spinal cord neurons in CA samples and muscle-associated transcripts in PA preparations). For this reason, we implemented stringent GFP+ versus GFP− filtering (now presented as Fig. 4) to refine Nav1.8-enriched transcripts and to guide downstream validation experiments.

We have extensively revised the Results section "*Central and peripheral DRG axons have distinct translatomes,*" corresponding to Fig. 2, and the Discussion to more cautiously frame functional interpretations derived from the inclusive dataset, particularly where enriched transcripts may reflect shared tissue background. Statements implying postsynaptic remodeling capacity have been softened to reflect transcript enrichment rather than inferred functional activity. Importantly, the central conclusion that CA and PA harbor distinct translatomic profiles remains robust and is supported by both the inclusive and filtered datasets. See results and discussion. See Results section from pg. 3; line 26 – pg. 4; line 21, and Discussion section from pg. 9; lines 21-33 and pg. 11; lines 43-45.

3. Mechanism of SFPQ asymmetry
The finding that SFPQ is selectively enriched in peripheral axons is highly intriguing. Given its central role in the authors' model, it would strengthen the Discussion to elaborate on potential mechanisms governing the asymmetric localization of SFPQ itself.

We thank the reviewer for this suggestion. We have expanded the Discussion to briefly consider potential mechanisms underlying SFPQ asymmetry, including differential motor engagement and branch-specific cytoskeletal organization, such as microtubule post-translational modifications that influence kinesin-dependent transport. We now say "One possibility is differential motor engagement or retention of SFPQ-containing RNP granules, potentially influenced by branch-specific cytoskeletal organization, including differences in microtubule post-translational modifications that regulate kinesin-based transport [88,89]. Defining how this asymmetry is established will be an exciting area of future investigation" on pg. 11; line 13.

Reviewer #3 (Remarks to the Author):

The authors used a TRAP-seq approach with Nav1.8-Cre x L10aGFPfl/fl mice to identify mRNAs translated in peripherally vs. centrally projecting DRG axons in 1 week old mice. Surprisingly, there are different RNA populations in the peripheral vs. central axons of these unipolar neurons. They identify the RNA binding proteins SFPQ and SRSF10 as enriched in peripheral vs. central axons based on motif enrichment across the translatome datasets and use IP to identify enriched binding for mRNA regulons by these proteins. Overall, the study is very well-done and provides a key scientific advance for understanding how molecular identities of subcellular regions/domains can be crafted by RNA transport and local translation. Ribosome profiling used here is a strength of the study since it gets past just the presence of the RNA and shows translation or at least ribosome interaction of the mRNAs. The authors provide validation of compartment selective translation for some identified mRNAs using puro-PLA type experiments. I only have a few comments, most of which can be settled with clarification in the text or modifications.

We thank the reviewer for these kind comments. The reviewer raised important questions. Therefore, rather than simply modifying the text, we have carried out additional experiments to address these issues.

Major points:

1. It is puzzling that the authors use 1 week old mice and infer physiological functions that we typically do not consider at that age – i.e., pain and neuropathy as they cross reference with paclitaxel transcriptomics

work. I suspect there were technical reasons for using young mice DRGs, but I think the authors should make that clear to the readers and clarify whether there would be developmental differences expected.

We thank the reviewer for this important point. Early postnatal (P3–P7) mice were intentionally used to reduce myelination and glial abundance in sciatic nerve and spinal cord preparations. At this stage, axons are less heavily myelinated and there are fewer mature glial cells, which improves RNA yield and enhances neuronal specificity in TRAP-seq experiments. In addition, the higher levels of axonal translation seen early in life enable more rigorous analysis. These points are now clarified in the Methods section on pg. 13; lines 19-22.

We feel that this is an important question that we should also address experimentally. Therefore we have now examined adult (2-month-old) mice and assessed local translation of selected ion channels (5-HTR3A, HCN1, NAV1.2, NAV1.9) using puromycin labeling in axonal compartments. These data confirm that axonal translation of key targets is maintained in mature animals and that there continues to be similar selectivity (see revised Figures 6 and Supplementary Figure S4).

2. Considering # 1, is the cross-dataset comparison between this study's TRAP dataset and the single-cell RNA-Seq dataset of DRG neurons treated with paclitaxel a fair comparison since adult mice and only somas (i.e., not central or peripheral axons) were used for the paclitaxel study? The authors need to point out this limitation of the study and the complications that my result from that.

We agree that this is an important point. Our goal in this analysis was to address questions regarding the functional significance of the axon-selective translatomes. We postulate that localized translation facilitates responses to external stimuli. Therefore, we wanted to see if the axonal translatome includes components that change in response to a critical stimulus known to affect sensory neurons such as chemotherapy. While the paclitaxel dataset was generated from adult DRG soma using single-cell RNA-seq and does not resolve axonal compartments, this comparison allowed us to identify locally translated genes that might be involved in the response to chemotherapy. We have revised the manuscript to explicitly acknowledge this limitation in the Discussion on pg 10; lines 47-54, and have tempered language implying direct functional consequences (see response to Major Point #1).

In addition, to more directly assess changes in the axonal translatome in response to paclitaxel, we have now examined paclitaxel-treated adult mice and evaluated changes in the local translation of Htr3a, an ion channels that we identified and verified as being locally translated, and that was previously shown to change in response to chemotherapy (see revised Fig. 6). Notably these new experiments provide new evidence that axonal translation of serotonin receptors changes in response to neuropathic stimuli. We thank the reviewer for this important comment, and we are very pleased to find that there are indeed changes in translation of Htr3a in the central axons in response to paclitaxel. These new data are in Figure 6, and we believe that this has improved the study.

I think they also need to back down statements like in the summary where they state encoded proteins 'confer distinct electrophysiological, synaptic, and regenerative properties' – statements in the discussion are also overreaching their data.

We appreciate this comment and agree that certain summary statements could be interpreted as implying functional validation beyond the scope of our data. We have revised the Abstract and Discussion to moderate this language and use wording that reflects association with, rather than direct demonstration of, these functional programs. These revisions ensure that the conclusions remain aligned with the transcriptomic and translational evidence presented. In the abstract, we now say "may confer" and in addition we have adjusted language throughout the manuscript abstract, results, and discussion sections.

3. In supplemental figure 3, several ion channels were identified that were locally translated in peripheral axons. The authors suggest that the local translation of these ion channels may modify pain responses. Is there a way to show this in vivo with knockdown studies?

We agree that in vivo perturbation of axonally translated ion channels would be an important next step to directly assess their contribution to sensory physiology. The focus of the present study was to define compartment-specific translatomes and identify candidate molecular programs underlying axonal specialization. We have revised the Discussion to more clearly frame these ion channels as candidates for future functional investigation in vivo and we look forward to such studies.

4. The comparing motifs across the peripherally vs. centrally enriched TRAP-seq data sets is a clever approach to get at potential compartment selective RBPs. The authors refer to these as 'binding sites' later in the text. I think they need to be clear that those are potential or putative binding sites as their pull down data do not address specific sites of binding (unless I missed that detail).

We agree with the reviewer and have revised the manuscript to clarify that the identified motifs represent predicted or putative RBP binding sites. Our RIP-seq experiments confirm association between RBPs and target transcripts but do not resolve specific binding sites within the 3′UTR. We have adjusted the terminology throughout the Results section, "*RBPs are positioned to orchestrate the subcellular localization of axonal translatomes,"* from pg. 7; lines 8-42, to avoid implying direct site-level validation.

5. For Figure 6, the Nav1.8-tdT images for A look more punctate and not continuous like the Nav1.8-tdT labeled axons in panels B & C. The sciatic nerve panel for Nav1.8-tdT in panel A looks like it is showing Schwann cell nuclei.

We thank the reviewer for this observation. Upon re-examination, we agree that the Nav1.8-tdTomato signal in Fig. 6A did not optimally represent continuous axonal labeling and may have included non-axonal structures. We replaced this panel with a different representative image that more clearly demonstrate axonal localization. The revised figure is now included in the manuscript.

Minor points:

1. On line 51, the authors reference Campbell et al (2001) and a recent review from the Sotelo group to emphasize that ribosomes are present in and mRNAs translated within axons. Notably, Zheng et al's publication on ribosomes in DRG axons (J Neurosci 21, 9291) was published a few weeks earlier than the Campbell paper. The authors should reference both of these here.

We thank the reviewer for pointing this out. We have now added the Zheng et al. (J Neurosci, 2001) reference alongside Campbell et al. to reflect the timeline of early evidence demonstrating ribosomes in DRG axons.

2. For clarification, how far away from the soma were the sciatic nerves and dorsal roots collected? Should considerations be made for this distance since there may be differences between translation at the distal ends versus closer to the soma?

We thank the reviewer for this clarification request. Dorsal roots and sciatic nerves were transected at their junction with the ganglia. The central compartment consisted of the dorsal roots extending into the spinal cord, and the peripheral compartment consisted of the sciatic nerve extending distally to approximately the knee. Therefore, the peripheral axon samples represent proximal-to-mid axonal segments rather than distal terminals. We agree that translation may vary along the length of the axon, and our dataset reflects an aggregate axonal translatome within each branch rather than strictly distal or terminal-specific translation. We have clarified this point in the Methods on pg. 13; lines 25-27.

3. For line 344-6, sentence indicating that SFPQ IF is consistent with their prediction for enrichment in peripheral axons – I thought the senior author's lab had previously published selective peripheral localization of SFPQ from their Bclw work? If so, they should reference that here or somewhere in this section.

We had seen this previously and included it in our oral presentations, however it was never published in a peer reviewed study. We do now reference our previous studies on SFPQ regulons on pg. 7; lines 41-42.

4. For Figures 5 and 6, the authors need to indicate in the figure that the merged images include DAPI.

We thank the reviewer for this comment. We have revised the figure legends for new Figures 7 and 8 (corresponding to old Figures 5 and 6) to clearly indicate that the merged images include DAPI staining.

Authors Response to Reviewer Comments

We were very pleased with the positive comments from reviewers. The comments from reviewers are included below, with our responses in red.

Reviewer #1 (Remarks to the Author):

All my comments are addressed in the revised manuscript

We thank the reviewer for this positive comment about our revisions.

Reviewer #2 (Remarks to the Author):

The revised manuscript presents all key datasets as main figures, which are arranged in a way that makes it easier to follow the flow of ideas leading to the main conclusion of this study that DRG neurons have a mechanism that enables axon branch-specific mRNA transport. The datasets are presented in a way that acknowledges key limitations of the technology (i.e., the background of immunoprecipitation), which are rigorously monitored by matching experimental controls. The discussion is balanced and cautious. This manuscript tackles an important question in neuronal cell biology with a cutting-edge technique, and the conclusions and datasets will be of interest to the wide audience of Nature Communications. I support the publication of the revised manuscript.

We thank the reviewer for these kind comments about our study and recent revisions.

Reviewer #3 (Remarks to the Author)

The authors have addressed all of my and (I think) the other reviewers' concerns. The addition of adult DRG neuron data strengthens the manuscript and the authors have included limitations of their data that will be important for the reader. I think this manuscript represents an advance for the field and should be an important technical resource. I only have 2 minor comments:

1. Pg 4, line 21 – concluding sentence is missing a period.

2. Pg 3, lines 18-20 – the authors refer to 'higher expression' of axonally translated genes. They should refer to this as higher axonal mRNA levels or axonal translation in the peripheral compartment.

We thank the reviewer for these kind comments about our revisions.

We have revised the minor comments:

1. We included a concluding period on Pg 4, line 21.
2. We have edited Pg 3, line 20 to say that all genes mentioned have "higher axonal mRNA levels" rather than higher expression.
